# Effect of chyme viscosity and nutrient feedback mechanism on gastric emptying

**DOI:** 10.1016/j.ces.2017.05.048

**Published:** 2017-11-02

**Authors:** Thomas E. Moxon, Philippe Nimmegeers, Dries Telen, Peter J. Fryer, Jan Van Impe, Serafim Bakalis

**Affiliations:** a Department of Chemical Engineering, University of Birmingham, Edgbaston, UK; b Department of Chemical Engineering, BioTeC+ & OPTEC, KU Leuven, Belgium

**Keywords:** Gastric viscosity, Nutrient feedback mechanism, Gastric emptying, Gastric secretion, Mathematical modelling

## Abstract

A comprehensive mathematical model of the digestive processes in humans could allow for better design of functional foods which may play a role in stemming the prevalence of food related diseases around the world. This work presents a mathematical model for a nutrient based feedback mechanism controlling gastric emptying, which has been identified *in vivo* by numerous researchers. The model also takes into account the viscosity of nutrient meals upon gastric secretions and emptying. The results show that modelling the nutrient feedback mechanism as an on/off system, with an initial emptying rate dependent upon the secretion rate (which is a function of the gastric chyme viscosity) provides a good fit to the trends of emptying rate for liquid meals of low and high nutrient content with varying viscosity.

## Highlights

• Gastric emptying rate linked to intestinal bioaccessability by feedback mechanism.
• Gastric secretion model links the secretion rate to the gastric viscosity.
• Model fits emptying of low and high viscosity liquid meals.

## 1 Introduction

Numerical modelling of the digestive system has been carried out from both a pharmacokinetic (Di Muria et al., 2010, Peng and Cheung, 2009, Stoll et al., 2000, Yu et al., 1996), and a food science perspective (Bastianelli et al., 1996, Dalla Man et al., 2006, Logan et al., 2002, Moxon et al., 2016, Penry and Jumars, 1986, Penry and Jumars, 1987, Taghipoor et al., 2012, Taghipoor et al., 2014). The general approach is to break the digestive system into compartments which can be described as ideal reactors. The stomach is typically described as a Continuous Stirred Tank Reactor (CSTR), whereas the small intestine has been described as a single CSTR, multiple CSTRs in series, or as a Plug Flow Reactor (PFR). Most of these models take only the dosage of the nutrient or drug into account when modelling the absorption, ignoring the physical properties of the meal, such as viscosity, and the interactions with the digestive system or other meal components. Here we will present a simple model to describe the influence of viscosity upon gastric processes (e.g., Marciani et al., 2001, etc.) and the effect of the nutrient based feedback mechanism upon gastric emptying (e.g., Brener et al., 1983, etc.). The aim is to develop a model which takes into account physical and chemical properties of the meal and can provide a greater understanding of food digestion. This could help in the development of functional foods to combat diet related diseases, such as obesity and type-2 diabetes, which are becoming increasingly more prevalent in modern society (Popkin, 2006, Jew et al., 2009).

The presence of a nutrient based feedback mechanism, also referred to as ’duodenal brake’, has been observed by numerous researchers (Brener et al., 1983, Calbet and MacLean, 1997, McHugh and Moran, 1979, Shahidullah et al., 1975), by measuring gastric emptying rate with intraduodenal nutrient secretions. This mechanism allows for the pyloric sphincter to control the emptying of gastric content into the duodenum depending upon the amount of nutrient already present in the proximal small intestine, ensuring a constant rate of calories per minute entering the small intestine, and the nutrient type (Calbet and MacLean, 1997). The sensing of nutrients in the intestine is carried out by taste receptors, similar to those found in the mouth and nasal cavity, e.g., the T1R family of receptors allows for the sensing of sugars (Depoortere, 2014, Young, 2011). The stimulation of these sensors induces the secretion of the hormone Cholecystokinin (CCK), which acts to decrease the gastric emptying rate and increase satiety (Depoortere, 2014), and/or slow gastric emptying via stimulation of the vagal nervous system (Young, 2011).

Whilst nutrient content will have an effect upon the gastric emptying rate other meal properties will also have an influence. The volume of a meal has been shown to speed up gastric emptying (Hunt and Stubbs, 1975). The viscosity of the chyme can also have an effect upon the gastric emptying rate, with some experimental results showing higher viscosities increase the gastric emptying rate of nutrient meals (Shimoyama et al., 2007, Vist and Maughan, 1995), while others show the opposite (Yu et al., 2014, Marciani et al., 2001). For non-nutrient meals, it has been shown that the gastric volume shows little variation with the viscosity of the meals input (over a 1000 times increase in zero shear viscosity), but that the level of secretions will be much greater with higher viscosities - resulting in large drops in the viscosity of the chyme (Marciani et al., 2000).

This work will build upon a model previously developed in Moxon et al. (2016). The aim is to demonstrate the viscosity of a liquid meal affects the mass transfer of nutrients within the intestine, and will influence the gastric emptying rate via a feedback mechanism. Further to this a model for the secretion of gastric juices is proposed, assuming the rate of secretion is influenced by the gastric chymes viscosity, and that the emptying rate previously assumed constant (γ) (Moxon et al., 2016), is affected by the meal and gastric properties. The work will attempt to fit model outputs to experimental data and gain numerical values for the constants used in the model from the experiments.

## 2 Model structure

**Fig. 1:**
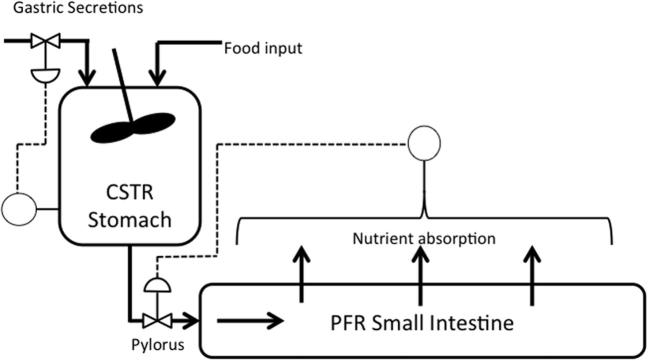
Schematic of the processes occurring in the stomach and small intestine which will be modelled. The absorption rate from the small intestine will control the opening of the pyloric sphincter, and the rate of secretions will be controlled via properties of the food in the gastric compartment.

The model presented builds upon previous work (Moxon et al., 2016), which assumed the stomach can be modelled as a continuous stirred reactor (i.e., fully mixed), and small intestine as a plug flow reactor and looked at how gastric emptying rate and intestinal lumen mass transfer rate can influence the absorption of nutrients. The model will link the gastric emptying rate and luminal mass transfer rate by introducing a nutrient based feedback mechanism observed from literature (Brener et al., 1983). Secretion in the stomach can be initiated via 3 different phases (Di Mario and Goni, 2014): a cephalic phase, due to anticipation of food; a gastric phase, due to the presence of food in the stomach; and an intestinal phase, via a feedback mechanism from the content of the small intestine. A secretion model will focus on how meal properties might affect the gastric phase of secretion (the phase inducing the highest volume of secretions (Di Mario and Goni, 2014)) and the influence of secretions upon the chyme viscosity, which will play a role in the gastric emptying of the meal. A schematic of the model is shown in Fig. 1.

### 2.1 Model development

The model presented will investigate liquid meals, with a mass of nutrient (${\mathrm{Stom}}_{N0}\;[g]$) entering the stomach at t=0. The stomach will be modelled as a continuous stirred tank reactor with the output emptying into the duodenum over the time period $t\in [0\text{,}t_{f}]$, where $t_{f}$ is the final measurement time. The mass of nutrient in the stomach is represented as ${\mathrm{Stom}}_{N}$:

(1) $$\frac{\partial {\mathrm{Stom}}_{N}(t)}{\partial t}=-\gamma \;{\mathrm{Stom}}_{N}(t)$$

(2) $${\mathrm{Stom}}_{N}(0)={\mathrm{Stom}}_{N0}$$

where γ is the gastric emptying rate in s^−1^. It is assumed that the meal is consumed rapidly and that negligible gastric emptying or dilution of gastric content will occur before the whole meal is consumed. This assumption is more relevant for low viscosity liquid meals, which are consumed more rapidly than high viscosity meals (Marciani et al., 2001). It should also be noted that this assumption may not be appropriate if modelling the consumption of a normal (multi phase) meal, and a gastric filling function linked to time may be more appropriate in such cases.

The mass of nutrients in the small intestine will be modelled in terms of a 1-D advection-reaction equation, assuming the limiting factor in the absorption of nutrients will be the mass transfer rate within the intestinal lumen. This approach has been taken by others when looking at drug or food absorption (Stoll et al., 2000, Logan et al., 2002). It was shown by Yu et al. (1996) to give a good description of the intestinal transit time, much better than assuming a single compartment, and similar to assuming 7 CSTR compartments. The mass of nutrient in grams $\left({\mathrm{SI}}_{N}(z\text{,}t)\right)$ will be modelled along the temporal domain and spatial domain, z∈[0,L], where *z* is the position along the length of the intestine in metres, and *L* is the total length of the small intestine (=2.85 m (Stoll et al., 2000, Barrett et al., 2005)), and position z=0 represents the position of the pyloric sphincter:

(3) $$\frac{\partial {\mathrm{SI}}_{N}(z\text{,}t)}{\partial t}=\left\{\begin{array}{cc} \gamma \;{\mathrm{Stom}}_{N}(t)-\overset{¯}{u}\;\frac{\partial {\mathrm{SI}}_{N}(z\text{,}t)}{\partial z}-K_{a}\;{\mathrm{SI}}_{N}(z\text{,}t) & \text{if}\;z=l_{0}\\ -\overset{¯}{u}\;\frac{\partial {\mathrm{SI}}_{N}(z\text{,}t)}{\partial z}-K_{a}\;{\mathrm{SI}}_{N}(z\text{,}t) & \text{Otherwise} \end{array}\right)$$

(4) $${\mathrm{SI}}_{N}(z\text{,}0)=0$$

With the following Neumann boundary conditions:

(5) $${\left(\frac{\partial {\mathrm{SI}}_{N}}{\partial z}\right|}_{z=0}={\left(\frac{\partial {\mathrm{SI}}_{N}}{\partial z}\right|}_{z=L}=0$$

The advection term $\overset{¯}{u}$ will be the mean velocity ($1.7\times 10^{-4}$ m/s (Stoll et al., 2000)). $K_{a}$ is the absorption constant of the nutrients in the intestinal lumen, linked in previous work to the mass transfer coefficient in the lumen (Moxon et al., 2016), but will be estimated from experimental data and as such will take into account the effect of radial transfer and mixing upon the absorption rate.

The mass entering at time, *t*, to the small intestine from the stomach will be assumed to enter as a spherical bolus of radius $l_{0}$, and enter at position $z=l_{0}$ along the intestine.

The absorption rate of nutrients from the intestinal lumen, will be modelled as the integral of the reactive terms from Eq. (3) over the length of the intestine:

(6) $$A(t)=\int_{z=0}^{L}K_{a}{\mathrm{SI}}_{N}\mathrm{dz}$$

### 2.2 Feedback mechanism

It will be assumed that the feedback mechanism is mediated by the bioaccessibility of the nutrient in the intestinal lumen (Depoortere, 2014), and that this controls the rate at which gastric chyme empties. Here the feedback mechanism is triggered by the rate of absorption, described by Eq. (6). From literature (Brener et al., 1983, Calbet and MacLean, 1997) it has been identified that the gastric emptying is controlled to ensure a constant rate of calories, and the model will assume the mechanism acts as an on/off switch, acting instantaneously. A maximum absorption rate, $A_{\max}$, will be set, and if this rate is exceeded the gastric emptying rate, γ, will be set to zero:

(7) $$\gamma =\left\{\begin{array}{cc} 0 & \text{if}\;A(t)>A_{\max}\\ \gamma_{0} & \text{otherwise} \end{array}\right)$$

To ensure smoothness Eq. (7) can be approximated to:

(8) $$\gamma =\gamma_{0}\left[1-\left(\frac{1}{1+\exp(\tau_{A}(A(t)-A_{\max}))}\right)\right]$$

The term $\tau_{A}$ is set to a value of $5\times 10^{6}$ g/s to ensure that the function behaves in the same way as the logical one (a physiological interpretation and greater understanding is left for future work).

### 2.3 Secretion model

Data on gastric chyme viscosity was taken from *in vivo* experiments by Marciani et al. (2000) which measured the gastric response to non-nutrient meals of different viscosities. Experiments were conducted for four fluids of different viscosities, and Echo-planar MRI was used to assess the volume remaining in the stomach and viscosity of the gastric chyme. Nasogastric tubes were also used to take samples from the stomach and measure the viscosity of gastric chyme.

The model will assume the gastric content is perfectly mixed and that secretions are a function of viscosity only. The viscosity of the meal will be assumed to be a function of the concentration of thickener present. The assumption of perfect mixing is more accurate for the low viscosity solutions than for the higher viscosity solutions, and for two phase meals it has been shown that the solid phase resides in the proximal stomach for long periods of time, when compared to liquid phases (Collins et al., 1991), as such this assumption may not be applicable to a two phase meal. Results shown in Marciani et al. (2001) for the dilution of highly viscous meals highlight that dilution is much greater at the outer edge of the chyme bolus initially, with greater dilution towards the centre taking more time. This can also be seen via the variation in viscosity measurement by Marciani et al. (2000); for the high viscosity solution (initially 11 Pa s) the measured viscosity in the stomach (after 12 min) varied between 1 and 8.5 Pa s, whereas the variation was smaller for the consumption of 2 Pa s meal (0.9–2.0 Pa s). The assumption of perfect mixing will therefore lead to more accurate results when considering the emptying of low viscosity meals, but should still provide insight into the emptying of higher viscosity liquid meals. However when looking at solid components separate compartments may be required to account for the distribution between proximal and antral regions of the stomach.

The relationship between the concentration of locust bean gum (LBG) and viscosity was found from the initial measurements, and the fit carried out using MATLAB curve fitter application. The most reasonable fit was found using a power law, but normal meals are likely to have more complex rheological properties.

(9) $$\mu =a_{L}C_{\mathrm{LBG}}^{b_{L}}$$

where $a_{L}=2$ Pa s m^3^/g and $b_{L}=4.21\;[–]$.

The rate of secretions into the gastric compartment have been shown to increase with the viscosity of a meal (Marciani et al., 2000, Marciani et al., 2001), and that this could be due to the effect of gastric distension which has been shown to increase the secretion rate of gastric acid (Grötzinger et al., 1977). It is then assumed that the secretion rate will be a function of the viscosity of digesta in the stomach:

(10) $$K_{\sec}=\lambda_{s}\mu^{b}+S_{b}$$

where $\lambda_{s}$ and *b* are constants to be evaluated and $S_{b}$ is the basal secretion rate, i.e., that occurring with no stimulation.

To describe the gastric compartment two more components are added to the original gastric model (Eq. (2)), one for the mass of LBG in the meal (${\mathrm{Stom}}_{\mathrm{LBG}}$), to allow viscosity calculations, and one for the non-nutrient liquid (${\mathrm{Stom}}_{\mathrm{liq}}$), which will have an input from the secretions.

(11) $$\frac{\partial {\mathrm{Stom}}_{\mathrm{liq}}}{\partial t}=K_{\sec}(\mu )-\gamma \;{\mathrm{Stom}}_{\mathrm{liq}}(t)$$

(12) $$\frac{\partial {\mathrm{Stom}}_{\mathrm{LBG}}}{\partial t}=-\gamma \;{\mathrm{Stom}}_{\mathrm{LBG}}(t)$$

(13) $$C_{\mathrm{LBG}}=\frac{{\mathrm{Stom}}_{\mathrm{LBG}}(t)}{{\mathrm{Stom}}_{\mathrm{liq}}(t)/\rho_{w}}$$

where $\rho_{w}$ is the density of the non-nutrient liquid (assumed to have the properties of water). The total mass in the stomach will be:

(14) $${\mathrm{Stom}}_{\mathrm{tot}}={\mathrm{Stom}}_{N}+{\mathrm{Stom}}_{\mathrm{liq}}+{\mathrm{Stom}}_{\mathrm{LBG}}$$

Evaluating the secretion rate and viscosity of the gastric chyme allows the initial gastric emptying rate ($\gamma_{0}$ in Eq. (8)) to be evaluated as a function of the chymes properties. An initial rapid rate of emptying has been identified in literature and linked to the volume in the stomach (Brener et al., 1983, Moran et al., 1999), and the viscosity (Kusano et al., 2011, Marciani et al., 2001, Shimoyama et al., 2007). In the results of Marciani et al. (2000), varying the viscosity of non-nutrient meals resulted in negligible variability in gastric half life, but did increase the secretion rate.

To determine which of these factors are important in the initial rapid emptying phase, a number of different hypotheses for the dependence of the parameter $\gamma_{0}$ were tested. The following equations were defined to identify if the volume, viscosity or secretion rate or combination best describes the emptying:

(15) $$\gamma_{0}=m_{\mu }\mu +m_{s}V_{\mathrm{tot}}$$

(16) $$\gamma_{0}=m_{s}V_{\mathrm{tot}}+C_{1}$$

(17) $$\gamma_{0}=m_{\mu }\mu +C_{1}$$

(18) $$\gamma_{0}=m_{\sec}K_{\sec}+C_{1}$$

(19) $$\gamma_{0}=m_{\sec}K_{\sec}+m_{s}V_{\mathrm{tot}}$$

Here $V_{\mathrm{tot}}$ is the total volume in the stomach (${\mathrm{Stom}}_{\mathrm{tot}}/\rho_{w}$), μ is the gastric viscosity, and $K_{\sec}$ is the gastric secretion rate. $m_{\mu }$ [1/(Pa s^2^)], $m_{s}$ [1/(m^3^ s)], and $m_{\sec}$ [1/g] are the constants of gastric emptying as a function of the viscosity, gastric volume, and gastric secretion rate, respectively, and $C_{1}$ is a constant emptying rate independent of the three factors. For nutrient meals the overall emptying rate will depend upon the feedback mechanism (Eq. (8)).

A parameter estimation will be carried out using Eqs. (15), (16), (17), (18), (19) against experimental data from Marciani et al. (2000), and the Akaike Information Criterion (*AIC*) will be used to evaluate which of the models gives the best description of the experimental results (Burnham and Anderson, 2004):

(20) $$\mathrm{AIC}=n\ln\left(\frac{\mathrm{SSE}}{n}\right)+2p$$

where *n* is the number of experimental data points, *p* is the number of parameter to be fit and *SSE* is the sum of squared errors.

The model assumes that the reduction in the viscosity of the gastric content is due to the dilution of the thickener in the gastric compartment, though it should be noted that there will also be dilution due to saliva. The effect of digestive enzymes (salivary α-amylase and proteases) will also influence the viscosity of gastric chyme (De Wijk et al., 2004).

### 2.4 Methods

#### 2.4.1 Sensitivity analysis

To analyse how the model outputs vary with respect to the estimated parameters a sensitivity analysis was carried out. In this work a finite difference approach will be used to evaluate the sensitivity:

(21) $$\frac{\partial f}{\partial \theta_{i}}=\frac{f(\theta_{i}+\theta_{i}\varepsilon )-f(\theta_{i})}{\theta_{i}\varepsilon }$$

where *f* is the model output and $\theta_{i}$ is a parameter which is changing by a fractional perturbation of ε. To investigate the relative effect and compare systems with different input masses etc. the sensitivities will be normalised with the nominal value of the parameter and the input to the model for the different experiments ($f_{0}$). All sensitivities quoted will take the following general form:

(22) $$S^{*}=\frac{\partial f}{\partial \theta_{i}}\frac{\theta_{i}}{f_{0}}$$

#### 2.4.2 Monte Carlo analysis

A Monte Carlo analysis will be carried out to assess the quality of the parameters estimated. This will involve applying random noise to the experimental measurements over 5000 iterations to assess the parameter estimates response to these changes. Histograms showing the distribution of the parameter values will be used to assess the quality of the estimates. The analysis will allow for the experimental noise to be included in the parameter estimates and allow calculation of variance in model parameters, which could be induced due to variation between people, or time of day (circadian cycle), etc.

## 3 Results and discussion

The system of equations were solved numerically in MATLAB using the forward Euler method to discretise in the temporal domain, and a backward finite difference method to discretise in the spatial domain, this is summarised in Appendix B.

This section presents and discusses the results of the models developed. Firstly the feedback mechanism (Eq. (8)) will be presented on its own, with parameter estimations against 3 different sets of experimental data, those of Brener et al., 1983, Calbet and MacLean, 1997, Vist and Maughan, 1995, a sensitivity analysis will be carried out on the estimated parameters followed by Monte Carlo analysis. Then the secretion model (Eqs. (9), (10), (11), (12), (13), (14), (15), (16), (17), (18), (19)) will be applied to a non-nutrient meal (Marciani et al., 2000), and parameters evaluated. Finally the two models will be combined and parameter estimation carried out using experimental data (Marciani et al., 2001), to show how both gastric secretions and nutrient feedback play an important role in the gastric emptying rate.

### 3.1 Feedback mechanism

#### 3.1.1 Parameter estimation

**Fig. 2:**
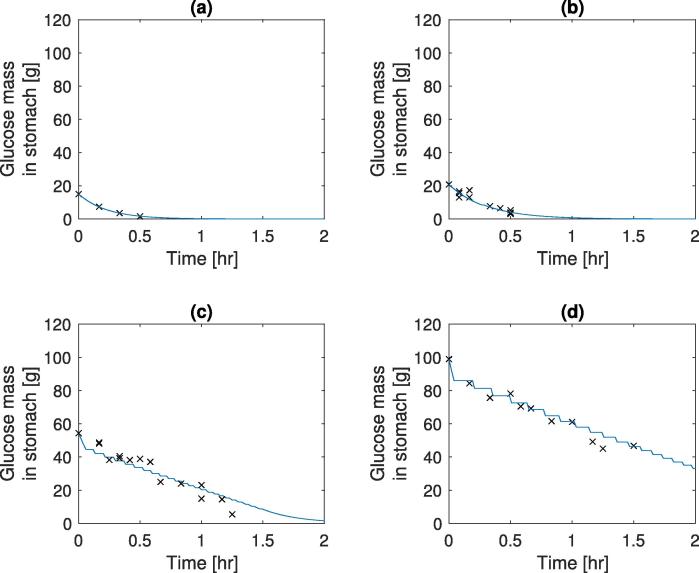
Model output & experimental results for emptying of different glucose solution from the stomach, solid lines represent the simulated results and dots represent experimental data. (a) 15 g initial mass (Calbet and MacLean, 1997), (b) 20 g initial mass (Brener et al., 1983), (c) 50 g initial mass (Brener et al., 1983), and (d) 100 g initial mass (Brener et al., 1983).

**Fig. 3:**
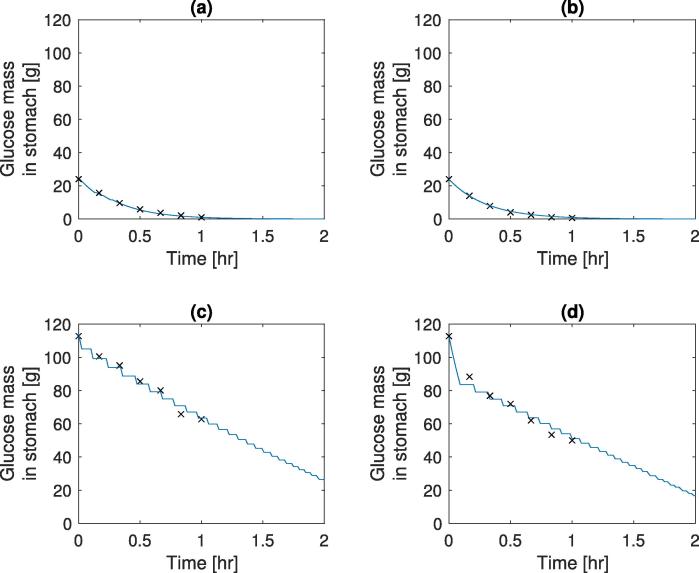
Model output and experimental results for emptying of different glucose solution from the stomach, with high and low polymer and glucose, solid lines represent the simulated results and dots represent experimental data from Vist and Maughan (1995). with initial masses (a) 25 g initial mass, low viscosity, (b) 25 g initial mass, high viscosity, (c) 112.8 g initial mass, low viscosity, and (d) 112.8 g initial mass, high viscosity.

A parameter estimation was carried out for $\gamma_{0}\text{,}\;A_{\max}$, and $K_{a}$, along with this the input mass (${\mathrm{Stom}}_{N0}$) was allowed to vary to take into account experimental noise at *t* = 0. The parameter estimations were carried out using the *lsqnonlin* function in MATLAB with experimental data from 3 different sources with different conditions (conditions and optimal parameter values are shown in Table 1). The model outputs (using model Eqs. (1), (2), (3), (4), (5), (6), (7), (8)), at optimal parameter values, and experimental results for the temporal change in gastric nutrient content after a meal has been consumed are shown in Fig. 2, Fig. 3. It is observed that in plots (a) and (b) in both figures, the emptying curve for the glucose solutions can be described as an exponential function of time. This is due to the mass of glucose in the small intestine not being sufficiently high to trigger the feedback mechanism for sustained periods of time with Figs. 2(b) and 3(a) triggering the mechanism for a short period of time around 15 min. Table 1 shows the fitted values for the emptying rate. The differences in values could be due to one of the following factors not taken into account in the current model: different volumes of the liquid meal (Hunt and Stubbs, 1975), different gastric secretion rates due to the composition of the meal (Marciani et al., 2000), different rheological properties (Marciani et al., 2001, Shimoyama et al., 2007), and variation between those tested, some of these factors (Viscosity and secretion rate) will be studied later.

**Table 1:** Estimated parameters for simulations of different experimental results, (HP - high polymer concentration).

	Conditions	${\mathrm{Stom}}_{N0}$ (*g*)	$\gamma_{0}$ (s^−1^)	$A_{\max}$ (g/s)	$K_{a}$ (s^−1^)	Experimental data
a	15 g input	15.03	$12\times 10^{-4}$	–	–	Calbet and MacLean (1997)
b	20 g input	20.82	$9.23\times 10^{-4}$	$7\times 10^{-3}$	$9\times 10^{-4}$	Brener et al. (1983)
c	50 g input	54.33				
d	100 g input	98.95				
e	24 g input	24.74	$9.22\times 10^{-4}$	$10\times 10^{-3}$	$1.7\times 10^{-3}$	Vist and Maughan (1995)
f	112.8 g input	114.28				
g	24 g input (HP)	23.98	$8.98\times 10^{-4}$	$10\times 10^{-3}$	$4.2\times 10^{-4}$	
h	112.8 g input (HP)	112.83				

Increasing the initial mass of glucose in the meal leads to an emptying which can be described as linear, after an initial rapid emptying period; this is observed both in the simulated and experimental data plots (c) and (d) in both figures. Unlike the data of plots (a) and (b), the mass of glucose in the small intestine increases to a level which is able to stimulate the feedback mechanism over longer periods of time, this leads to the step-like decrease in gastric content mass seen in the simulations (c) and (d) of Fig. 2, Fig. 3. The behaviour gives the constant emptying rate of calories described by Brener et al., 1983, Calbet and MacLean, 1997 amongst others. Due to the nature of the numerical solution the number of temporal discretisation points will have an effect upon the step like nature of the simulations.

#### 3.1.2 Sensitivity analysis

**Fig. 4:**
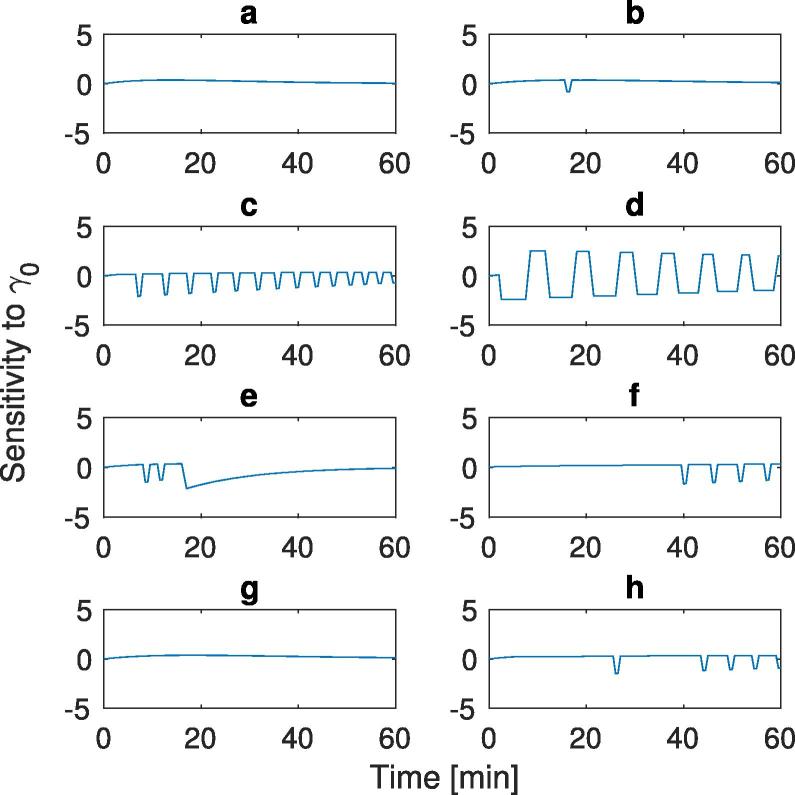
Sensitivity analysis of the 8 different experimental conditions shown in Table 1 with respect to the parameter $\gamma_{0}$. (a) 15 g initial mass, low viscosity, (b) 20 g initial mass, low viscosity, (c) 50 g initial mass, low viscosity, (d) 100 g initial mass, low viscosity, (e) 25 g initial mass, low viscosity, (f) 25 g initial mass, high viscosity, (g) 112.8 g initial mass, low viscosity, and (h) 112.8 g initial mass, high viscosity.

**Fig. 5:**
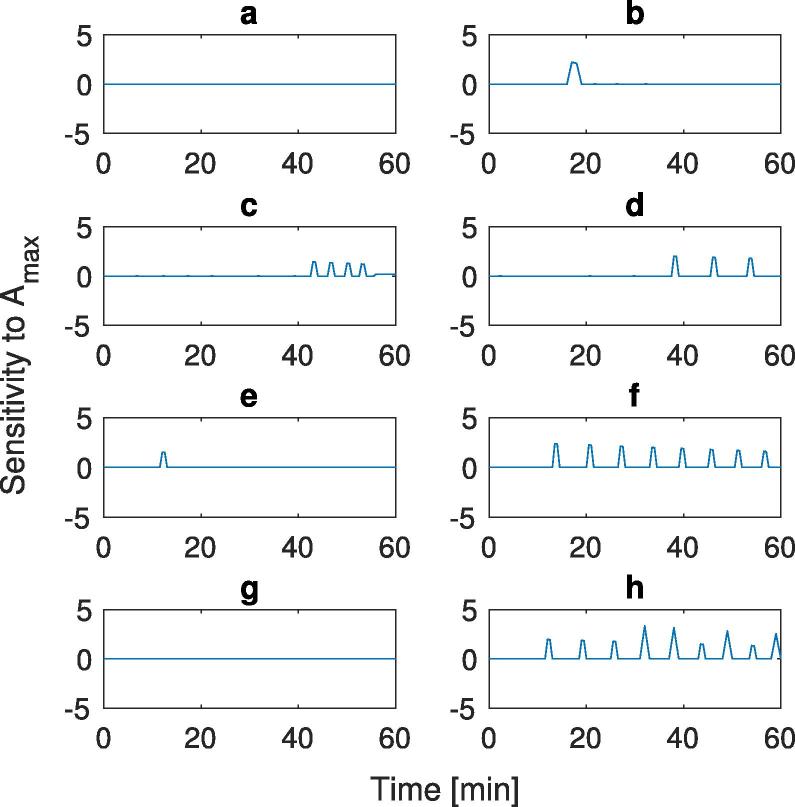
Sensitivity analysis of the 8 different experimental conditions shown in Table 1 with respect to the parameter $A_{\max}$. (a) 15 g initial mass, low viscosity, (b) 20 g initial mass, low viscosity, (c) 50 g initial mass, low viscosity, (d) 100 g initial mass, low viscosity, (e) 25 g initial mass, low viscosity, (f) 25 g initial mass, high viscosity, (g) 112.8 g initial mass, low viscosity, and (h) 112.8 g initial mass, high viscosity.

**Fig. 6:**
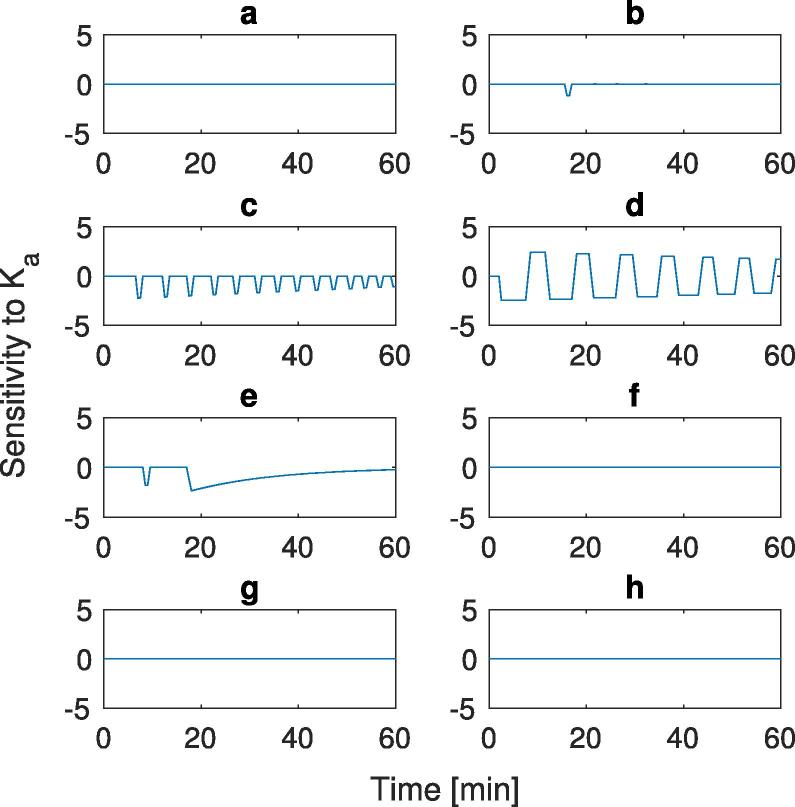
Sensitivity analysis of 8 different experimental conditions shown in Table 1 with respect to the parameter $K_{a}$. (a) 15 g initial mass, low viscosity, (b) 20 g initial mass, low viscosity, (c) 50 g initial mass, low viscosity, (d) 100 g initial mass, low viscosity, (e) 25 g initial mass, low viscosity, (f) 25 g initial mass, high viscosity, (g) 112.8 g initial mass, low viscosity, and (h) 112.8 g initial mass, high viscosity.

The effect of the parameters $\gamma_{0}\text{,}\;A_{\max}$, and $K_{a}$ upon the mass within the stomach postprandially was analysed using a sensitivity analysis. Small perturbations were applied to the parameters and the outputs compared using a finite difference approach. To do this data from Table 1 was used as the nominal values and a perturbation (ε) of 1% applied to the parameters. Fig. 4, Fig. 5, Fig. 6 show the sensitivity of the gastric content to the initial gastric emptying rate ($\gamma_{0}$) and the maximum absorption rate ($A_{\max}$), and intestinal lumen mass transfer rate ($K_{a}$), respectively. Plots (a)–(g) in each of the Figures correspond to the labels in Table 1 for the different input conditions for the meals.

The sensitivity of the emptying to the parameter $\gamma_{0}$ is shown in Fig. 4. The low nutrient simulations plots (a) and (g) do not initiate the feedback mechanism as the mass of glucose does not reach high enough levels in the small intestine to trigger the feedback mechanism, consequently the system empties exponentially with time. For plots (b) and (e), the feedback mechanism is initiated for a short period of time, seen from the spike in plot (b) and two spikes in plot (e) before the sensitivity plots return to behaving similar to those where the mechanism is not initiated (plots (a) and (g)), this is due to the glucose levels in the small intestine reach high enough levels to trigger the feedback mechanism. With the high nutrient meals (plots (c), (d), (f), and (h)), one can see a deviation from the zero point along with spikes occurring due to the feedback mechanism initiating, this is due to the bioavailability in the small intestine been maintained at a high level, this is seen later in plots (f) and (h) due to the higher viscosity meals taking longer to stimulate the feedback mechanism.

The sensitivity to parameter $\gamma_{0}$ is higher when the feedback mechanism is initiated. Increases in emptying rate could result in the subsequent increase in absorption rate that triggers the feedback mechanism and results in the increase in sensitivity. This is seen at the beginning of plot (e), where the increase in mass of nutrients due to the faster emptying rate (1% increase) triggers the feedback mechanism, and spikes are seen in the sensitivity plot. After around half the mass has emptied from the stomach the availability in the lumen will drop and the absorption rate will not reach the maximum again so the sensitivity reduces back to what would be expected during exponential emptying with time.

For low nutrient content meals the feedback mechanism is not initiated, hence the system will show no sensitivity to the parameter $A_{\max}$, which can be seen in Fig. 5, plots (a) and (g). As the amount of nutrient increases we start to see an effect. Looking at plot (b) we see a spike as the feedback mechanism initiates for a short amount of time before returning to zero, with plots (e) and (g) we see the effect of viscosity upon the sensitivity to $A_{\max}$ the high viscosity solution does not trigger the feedback mechanism, hence no sensitivity to $A_{\max}$ but the lower viscosity does lead to a peak in sensitivity and deviation from zero. At the higher nutrient contents (plots (c), (d), (f), and (h)) characteristic spikes can be seen due to the initiation of the feedback mechanism before falling back down to zero, hence the average rate of emptying will be maintained the same, with slight differences when the mechanism is initiated.

Fig. 6 shows the sensitivity to the absorption rate. This shows similarities to the sensitivity to $A_{\max}$, where for low glucose inputs (plots (a) and (g)) the stomach volume has no sensitivity to the absorption rate as the feedback mechanism is not initiated. This is also seen in plots (f) and (h), where the high nutrient content and high absorption rate means the feedback mechanism is initiated at the same point independent of the small perturbations in the absorption rate. Plots (b) and (e) show sensitivity similar to those for $A_{\max}$ where we see spikes before tending back to zero, and Plots (c) and (d) show sensitivity to the absorption rate similar to that of the $A_{\max}$ values. This is due to the rate being close to the maximum rate, so small changes mean the feedback mechanism is initiated at different points, resulting in a similar sensitivity profile to the $A_{\max}$ sensitivity.

#### 3.1.3 Monte-Carlo simulations

**Fig. 7:**
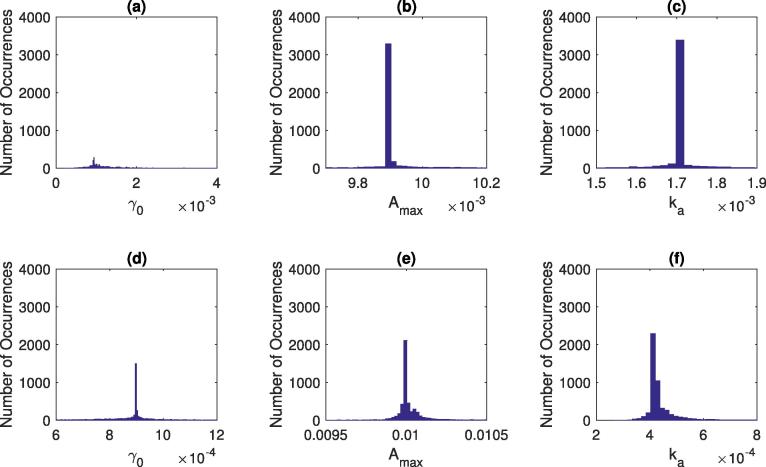
Parameter Histograms from Monte Carlo simulation with a total of 10,000 iterations using data from Vist and Maughan (1995), plots (a)–(c) for low viscosity solutions with high glucose levels (condition f), and plots (d)–(f) for high viscosity solutions with high glucose level (condition h).

The quality of the parameter estimations were analysed, and the parameter range defined using a Monte Carlo simulation. The data used for the simulation was from Vist and Maughan (1995), and two of the experimental conditions were chosen to analyse: (i) high glucose and low polymer (HGLP), Table 1 condition (f), and (ii) high glucose high polymer solutions (HGHP) Table 1 condition (g). These two data sets were chosen to ensure the feedback mechanism is triggered during emptying (high glucose) and to highlight the effect of changes in mass transfer rate (low and high viscosity). Random noise was added to the experimental data. This noise was taken from the range of maximum deviation from the mean experimental results from the repetitions (see Table 2). The initial emptying rate ($\gamma_{0}$), the feedback point ($A_{\max}$) and the absorption rate ($K_{a}$) were estimated, with a total of 5000 iterations carried out. The results are plotted as histograms in Figs. 7, with plots (a)–(c) showing the parameter distributions for the low polymer solution, and plots (d)–(f) showing the plots for the high polymer solutions.

**Table 2:** Experimental standard deviation (Vist and Maughan, 1995).

Time (min)	10	20	30	40	50	60
Condition f	16.1	9.7	9.7	11.1	12.4	10.6
Condition h	9.7	6.0	9.7	14.3	16.6	16.1

It would be expected that parameters which are insensitive to the experimental noise will show little variation across the iterations. This would be shown through a high number of occurrences at the same value. For parameters which are sensitive to the experimental noise, one would expect to see a distribution of values.

From Fig. 7 plots (b) and (c) it can be seen that there is little variation in the parameter estimates, indicting the estimations are insensitive to the experimental noise.

For parameter $A_{\max}$ (plot (b)), the results can be compared with the sensitivity analysis, Fig. 5 plot (f). Small perturbations in the parameter manifest in slight changes in when the feedback mechanism initiates, and the gastric emptying stops, but quickly equalises back to the control state, as the difference in the amount of time gastric emptying is suppressed is small.

We can postulate that for low viscosity high glucose meals, when the glucose enters the small intestine it will be in amounts which will initiate the feedback mechanism very shortly after consumption and that changes in the value have little effect upon the emptying rate. This is also seen for the value of $K_{a}$ (plot (c)).

Plot (a) showing the distribution of the $\gamma_{0}$ estimations shows a more Gaussian distribution than the insensitive parameters $A_{\max}$ and $K_{a}$, indicating a greater sensitivity to the experimental noise.

The high viscosity values behave differently when experimental noise is introduced. The meals will take longer to initiate the feedback mechanism due to the reduced bioaccessibility of intestinal nutrients. Reduced bioaccessibility results in a lower absorption rate, which is closer to the maximum rate ($A_{\max}$), therefore in Fig. 5 the spikes occur at a greater frequency than in the lower viscosity higher nutrient meals; hence plots (d)–(f) shows a more Gaussian distribution of the parameters values from the Monte Carlo simulation.

The estimated values for the parameters could be influenced by phenomena which have not been considered in the model so far. The effect of secretions upon the viscosity of the meal is not considered; it is expected that due to these secretions the viscosity will be dynamic, changing over time. The effect of secretions is likely to have a greater impact upon the high polymer solutions, as high viscosity meals have been shown to stimulate greater rates of secretion (Marciani et al., 2000). This is looked at in greater detail in the following section.

### 3.2 Non-nutrient meal secretions

Using data from Marciani et al. (2000) a model selection was carried out to determine which of the Eqs. (15), (16), (17), (18), (19) best describes the experimental results when used along with Eqs. (9), (10), (11), (12), (13) to describe gastric processing, using the *lsqnonlin* function in MATLAB. The objective function used was the sum of the squared differences between viscosity values (normalised by the initial viscosity) and the half time of gastric emptying for all 4 sets of experimental data (shown in Eq. (23), where *i* is the experimental data set (total *n*), and *j* is the sampling points (total *m*). $\mu_{0\text{,}i}$ and ${\mathrm{liq}}_{0\text{,}i}$ are the initial viscosity and liquid load for each experiment, and subscripts *exp* and *sim*, represent experimental data and model output, respectively). The Akaike Information Criterion (AIC) was calculated for each of the models and used to compare them.

(23) $$\mathrm{Obj}=\overset{n}{\underset{i=1}{\sum }}\overset{m}{\underset{j=1}{\sum }}{\left(\frac{\mu_{\exp\text{,}i\text{,}j}-\mu_{\mathrm{sim}\text{,}i\text{,}j}}{\mu_{0\text{,}i}}\right)}^{2}+\overset{n}{\underset{i=1}{\sum }}2\left(0.5-\frac{{\mathrm{Stom}}_{\mathrm{tot}}{|}_{t=t_{1/2\text{,}i}}}{{\mathrm{liq}}_{0\text{,}i}}\right)$$

Eqs. (17), (18) describe the experimental data much better than the other equations. Comparing the likelihood ($\exp(({\mathrm{AIC}}_{\min}-{\mathrm{AIC}}_{i})/2)$), the model utilising Eq. (17) describing the emptying as a function of viscosity is 0.9 times as likely as Eq. (18). But as Eq. (18) takes into account both the effect of viscosity and the change in volume (due to secretion) this model was chosen as a more physiologically relevant approach and will be used for further work.

**Fig. 8:**
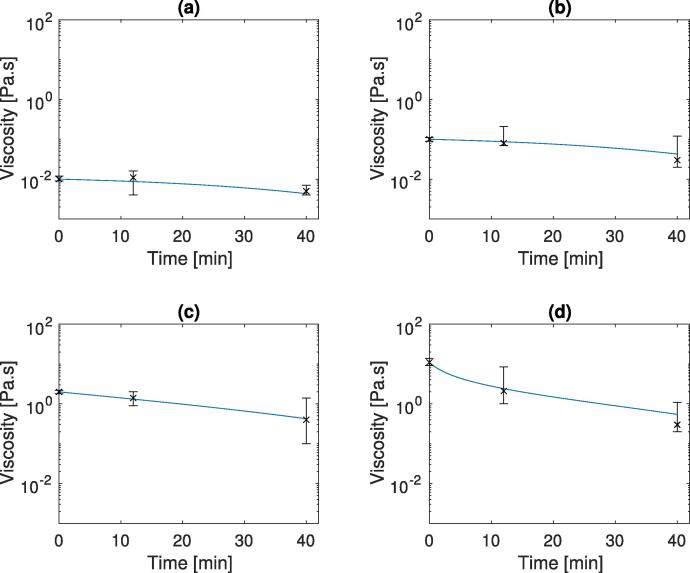
Viscosity profiles for 4 different input viscosities. Solid line shows model output (using Eq. (18)), crosses and error bars show the values from literature *in vivo* data (Marciani et al., 2000).

**Fig. 9:**
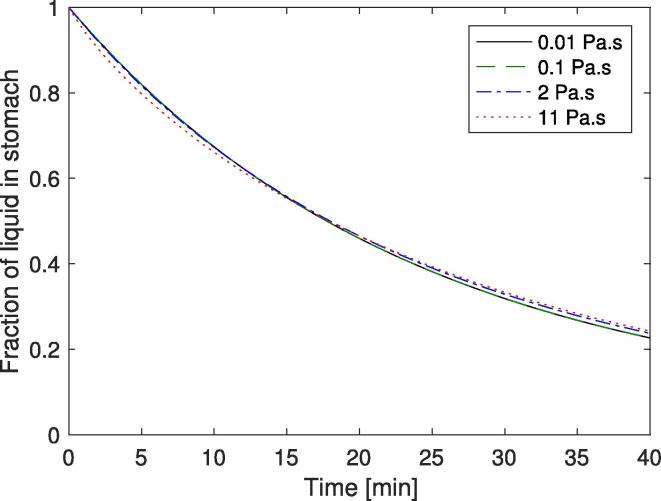
Volume profiles for 4 different input viscosities.

The parameter values for the optimised model using Eq. (18) to describe the initial gastric emptying rate (Eq. (8)) are shown in Table 3. The results of the optimal solution are shown in Fig. 8. This shows the simulated and experimental zero shear viscosity measurements from the gastric region. The values all show good fit to the experimental results and fall within the experimental variation. The higher viscosity solutions cause greater secretion rates, which in turn cause a greater reduction in the chyme viscosity. Fig. 9 also shows that the normalised stomach volumes have similar gastric half times for all four solutions, corresponding to what was seen in the *in vivo* work (Marciani et al., 2000).

**Table 3:** Optimal parameter values for non-nutrient meal secretion with the Upper and lower bound for parameter estimations calculated from 5000 iteration Monte Carlo simulation.

	*m*	$C_{1}$	$\lambda_{S}$	*b*	$S_{b}$
$\overset{¯}{\theta_{i}}$	0.0025	$6.58\times 10^{-4}$	0.018	1.5	0.018
$\overset{¯}{\theta_{i}}+2\sigma_{i}$	0.0174	$8.38\times 10^{-4}$	0.0621	2.2	0.028
$\overset{¯}{\theta_{i}}-2\sigma_{i}$	$3.27\times 10^{-4}$	$4.69\times 10^{-4}$	0.0014	0.2	0.0014

Using the parameter values from Table 3 as an initial guess, a Monte Carlo simulation was carried out using random experimental data points taken from the values between the extrema of the experimental variability. From this the variance in the parameter values over 5000 iterations were calculated. Using this variance an upper and lower limit for further parameter estimation was defined as $\overset{¯}{x}\pm 2\sigma$, to take into account 95% of the values estimated from the Monte Carlo simulation. These bounds are shown in Table 3.

### 3.3 Nutrient meal with secretions and feedback

The feedback model (Eq. (8)) and secretion model (Eqs. (9), (10), (11), (12), (13), (14), (15), (16), (17), (18), (19)) were then combined to investigate the effect of secretion and nutrient feedback mechanism upon gastric emptying of a liquid nutrient meal. Experimental data points were taken from Marciani et al. (2001), which studied the effect of viscosity on emptying of nutrient meals. Eq. (9) was used to predict the change in gastric viscosity with concentration. Although the experimental meals contained different sources of nutrient (63% lipid and 27% carbohydrate), it was assumed that the nutrients behaved the same and did not require enzymatic hydrolysis to be absorbed through the gut epithelium. The nutrient meals had a calorific content of 323 kcal and the control meals had a calorific content of 64 kcal.

A random initialisation used to obtain model parameters values with the upper and lower bounds of the parameter estimations chosen from the mean of the parameter values from the non nutrient meal plus/minus 2 times the standard deviation ($\overset{¯}{\theta_{i}}\pm 2\sigma_{i}$) from Table 3. The parameter value of $K_{a}$ was assumed constant along the length of the intestine (*in vivo* this will likely change due to intestinal secretions and mixing induced by intestinal wall motility) and estimated from the experimental results. To ensure the stability of the system a finer temporal step was required compared to the feedback only model; and in the current form of the model implies a faster feedback response. This was due to the stiffness of the modified equations; i.e., the parameter values are different orders of magnitude in size, and hence due to the chosen discretisation scheme smaller step sizes are required.

Due to the variability between different people etc. each data set was run separately, with the value utilised for $K_{a}$ consistent for each viscosity value.

**Fig. 10:**
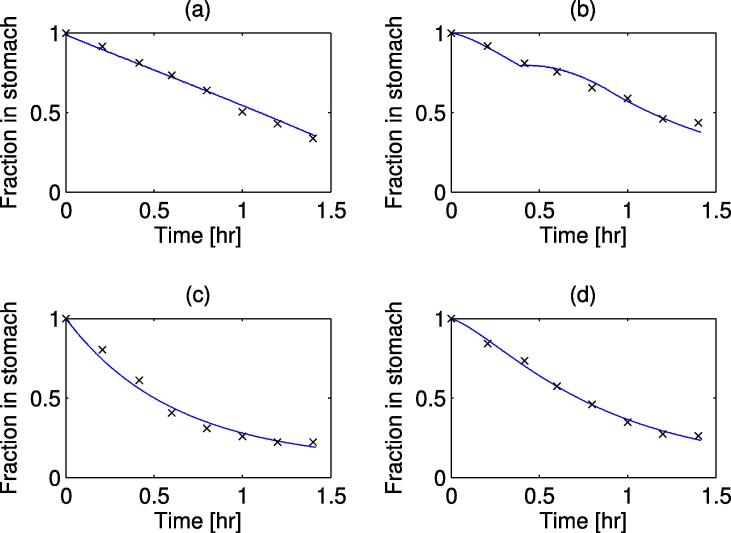
Gastric content after meal normalise against input volume, for (a) low viscosity nutrient meal, (b) High viscosity nutrient meal, (c) low viscosity control meal, (d) high viscosity control meal. solid line representing simulated results and crossed *in vivo* data (Marciani et al., 2001).

The optimal parameter values for the different meals are shown in Table 4, and Fig. 10 shows the gastric content against time for the different meals:

• The low viscosity nutrient meal (plot (a)) initiates the feedback mechanism and empties in a linear fashion.
• For the high viscosity meal (plot (b)) there is an initial lag period where little change in the gastric content occurs due to the high level of secretions. This is followed by a more linear emptying period until the feedback mechanism initiates and a slight plateau is seen. The plateau can be explained by the reduction in bioaccessibility (lower mass transfer rate) leading to higher nutrient concentration in the lumen before the feedback mechanism is initiated.
• The control meals, with low nutrient content, do not initiate the feedback mechanism, and the emptying rate is controlled by the viscosity and secretion rate (Eq. (18)).
  – The low viscosity control meal (plot (c)) shows a typical exponential emptying curve.
  – For the high viscosity control (plot (d)) a slight lag phase can be observed again at the beginning of the curve due to the higher rates of secretions.

**Table 4:** Optimal parameter values for different meals (Marciani et al., 2001), where LVN is low viscosity nutrient meal, HVN is high viscosity nutrient meal, LVC is low viscosity low nutrient meal, and HVC is high viscosity low nutrient meal.

Parameter	$A_{\max}$	$m_{\sec}$	$C_{1}$	$\lambda_{S}$	b	$S_{b}$	$K_{a}$
LVN	$1.04\times 10^{-2}$	$3.66\times 10^{-4}$	$6.77\times 10^{-4}$	$4.05\times 10^{-2}$	1.08	$0.57\times 10^{-2}$	$9.80\times 10^{-3}$
HVN	$0.98\times 10^{-2}$	$3.48\times 10^{-4}$	$4.74\times 10^{-4}$	$6.17\times 10^{-2}$	0.40	$1.51\times 10^{-2}$	$2.89\times 10^{-4}$
LVC	$1.00\times 10^{-2}$	$3.27\times 10^{-4}$	$4.69\times 10^{-4}$	$6.21\times 10^{-2}$	0.67	$2.13\times 10^{-2}$	$9.80\times 10^{-3}$
HVC	$1.01\times 10^{-2}$	$3.27\times 10^{-4}$	$5.86\times 10^{-4}$	$6.21\times 10^{-2}$	0.42	$2.34\times 10^{-2}$	$2.89\times 10^{-4}$

The parameter values estimated for these models all fall within the range found from the Monte Carlo simulation of the non nutrient meals (Table 3). The main difference in the model outputs are the result of changes in the value of parameter $K_{a}$. There is an order of magnitude difference in the value of $K_{a}$ for the low and high viscosity solutions. The assumption of the model is that the parameter will be a function of the mass transfer in the lumen and hence expected to change with viscosity of the meal (see previous work in Moxon et al. (2016)). There are smaller variations in other optimal parameter values. Parameters $b\text{,}\lambda_{S}$, and $S_{b}$ from Eq. (10) show some variability, with *b* having a lower value for the higher viscosity meals. This may result from the equation used to describe the viscosity values with LBG concentration (Eq. (9)). This equation was fitted from data in Marciani et al. (2000) with a maximum viscosity of 11 Pa s, whereas the high viscosity in the second data set (Marciani et al., 2001) had a viscosity closer to 30 Pa s, but no LBG concentration data. There could also be an additional mechanism stimulating the secretion other than the model proposed linked to the viscosity. The cephalic phase of secretion is not taken into account in this model, which would add secretions due to the anticipation of food and/or the sensing of nutrients in the mouth. This phase could explain the higher value of parameter *b* for low viscosity nutrient meal compared to low viscosity control meal, and may also be affected by the viscosity of the meal. The value $C_{1}$ also shows some variability which may imply other phenomena, as well as viscosity and volume change, which could influence the emptying rate.

**Fig. 11:**
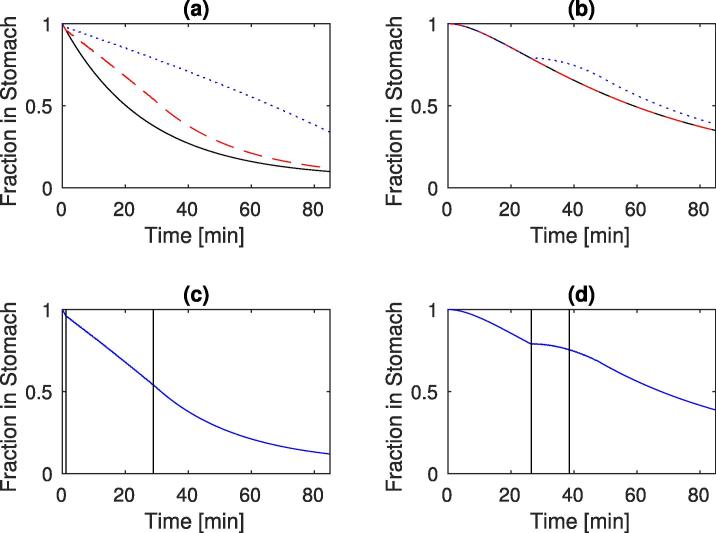
Predictions of Gastric responses for (a) low viscosity meals using parameters from LVN in Table 4, and (b) high viscosity using parameters from HVN in Table 4, for each set of parameters simulation was ran at initial glucose mass of 20 g (solid line), 40 g (dashed line), and 80 g (dotted line), (c) shows the 40 g low viscosity solution with vertical lines indicating when the feedback mechanism initiates and when it finally stops, (d) shows the 80 g high viscosity solution with vertical lines indicating when the feedback mechanism initiates and when it finally stops.

Fig. 11 shows simulations with (a) the optimal parameter values for the LVN from Table 4 for 3 different inputs of glucose: 20 g, 40 g, and 80 g, (b) is simulated with parameters for HVN from Table 4 for the same glucose inputs, (c) the 40 g simulation with LVN parameters with the initial feedback point and final feedback point marked with vertical lines, (d) same as (c) for the 80 g simulation from HVN parameters. In plot (a) the effect of the feedback mechanism can be seen. The 20 g curve does not initiating the mechanism, but the 40 g curve initiates the feedback almost straight away. This continues until around 30 min (highlighted in plot (c)) when the absorption rate drops (most of the glucose already absorbed), and is no longer high enough to stimulate the feedback mechanism, and the emptying returns to a more exponential pattern. The highest glucose solution (80 g) initiates the feedback mechanism and the curve follows a straight line over the whole 80 min simulation period.

For the high viscosity values in plot (b) neither the 20 g nor the 40 g solutions initiate the feedback mechanism and hence follow the same curve. The 80 g solution however does trigger the mechanism, just before 30 min, causing the emptying to slow. The absorption rate quickly drops to levels below that which would trigger the feedback mechanism and the emptying goes back to similar behaviour as the lower nutrient level solutions, this is highlighted in plot (d).

The ability to predict the temporal changes in the gastric viscosity could allow for better predictions of hormone release, such as *Gastrin* or *Ghrelin*, and be important in predicting a meals effect upon satiety, where more viscous meals reduce appetite (Marciani et al., 2000). Along with this, understanding the viscosity of intestinal chyme will allow better understanding of the secretion rate of incretins, which will be a function of intestinal nutrient bioaccessibility (Baggio and Drucker, 2007), and will play a role in the secretion of insulin.

**Fig. 12:**
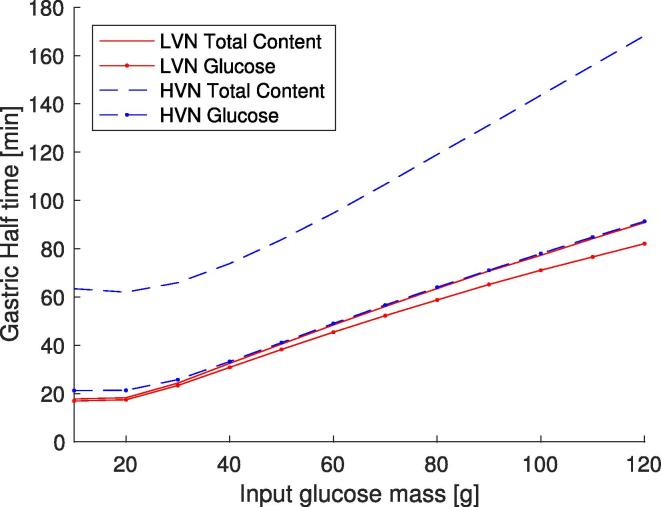
Prediction of the half time for the whole gastric content and the half time for the glucose input only were calculated at different initial glucose inputs, but constant volume. Parameters used in the model were taken from Table 4 for the optimal parameter values for the LVN and the HVN meals.

The effect of viscosity on the half emptying time was studied using the predicted parameters from Table 4 for the LVN and HVN meals, with the results shown in Fig. 12. The plots show the time for half the initial glucose content to empty for each set of parameters when different initial glucose loads are used with constant liquid volume, and the time for the gastric content to reach half the initial volume for the different glucose inputs. For all the plots, when the input mass of glucose is increased the half empty time also increases, with a larger difference in the half emptying time for the high viscosity meal compared to the lower viscosity meal. This is partly due to the increase in secretions expected with higher viscosity meals. This curve may have implications on the way gastric emptying is measured. Scintigraphy, for example, will label a particular component, e.g., glucose, and measure the amount in the gastric compartment, of this component only (Hveem et al., 1996). In contrast Marciani et al. (2000) use MRI, which will measure the entire gastric content. As such for high viscosity meals, the large volumes of secretions may lead to underestimation of the rate at which the nutrients (glucose in this case) are emptying.

## 4 Conclusion

The paper presents a mathematical model to describe the gastric emptying rate of nutrient liquid meals of varying viscosity (shown in Appendix A). To achieve this an attempt was made to model the nutrient initiated feedback mechanism present between the proximal small intestine and the pyloric sphincter The results indicate that with the estimation of two parameters: an initial emptying rate ($\gamma_{0}$) and a feedback cut off point ($A_{\max}$), the model can produce simulations to show the differing trends between low and high nutrient meals. This model was developed further to take into account the gastric secretions induced through meal viscosity and the subsequent effect on the parameter $\gamma_{0}$, this model predicted the increased secretion rate due to gastric chyme viscosity and subsequent rapid reduction in the viscosity values. The Monte Carlo analysis highlighted the variability in the parameter values which stem from the difference between individuals amongst other factors, which need to be considered when modelling the digestion of food. Including the model for gastric secretions and the influence on the emptying rate along with a nutrient feedback mechanism gave a model able to predict closely the gastric curves found for high and low nutrient meals of varying viscosity.

The models presented will go some way towards providing predictive capability for the emptying of viscous, nutrient-rich liquid meals, further work will look at validation of the absorption rate. Used in conjunction with models already available in literature for glucose-insulin system would allow for the prediction of postprandial plasma glucose curves and design of food tailored for different glycemic responses.

## Appendix A Secretion and feedback model

### A.1 Gastric compartment

Nutrient content in the stomach, over time period, $t\in [0\text{,}t_{f}]$

(1) $$\frac{\partial {\mathrm{Stom}}_{N}(t)}{\partial t}=-\gamma \;{\mathrm{Stom}}_{N}(t)$$

(2) $${\mathrm{Stom}}_{N}(0)={\mathrm{Stom}}_{N0}$$

Non-nutrient liquid:

(11) $$\frac{\partial {\mathrm{Stom}}_{\mathrm{liq}}}{\partial t}=K_{\sec}(\mu )-\gamma \;{\mathrm{Stom}}_{\mathrm{liq}}(t)$$

Thickener:

(12) $$\frac{\partial {\mathrm{Stom}}_{\mathrm{LBG}}}{\partial t}=-\gamma \;{\mathrm{Stom}}_{\mathrm{LBG}}(t)$$

Thickener concentration:

(13) $$C_{\mathrm{LBG}}=\frac{{\mathrm{Stom}}_{\mathrm{LBG}}(t)}{{\mathrm{Stom}}_{\mathrm{liq}}(t)/\rho_{w}}$$

Emptying rate:

(8) $$\gamma =\gamma_{0}\left[1-\left(\frac{1}{1+\exp(\tau_{A}(A(t)-A_{\max}))}\right)\right]$$

Initial emptying rate:

(18) $$\gamma_{0}=m_{\sec}K_{\sec}+C_{1}$$

Secretion rate:

(10) $$K_{\sec}=\lambda_{s}\mu^{b}+S_{b}$$

Viscosity:

(9) $$\mu =a_{L}C_{\mathrm{LBG}}^{b_{L}}$$

### A.2 Small intestine compartment

Only nutrient content will be modelled in the Small intestine compartment, the model will be over the spatial domain z∈[0,L]:

(3) $$\frac{\partial {\mathrm{SI}}_{N}(z\text{,}t)}{\partial t}=\left\{\begin{array}{cc} \gamma \;{\mathrm{Stom}}_{N}(t)-\overset{¯}{u}\;\frac{\partial {\mathrm{SI}}_{N}(z\text{,}t)}{\partial z}-K_{a}\;{\mathrm{SI}}_{N}(z\text{,}t) & \text{if}\;z=l_{0}\\ -\overset{¯}{u}\;\frac{\partial {\mathrm{SI}}_{N}(z\text{,}t)}{\partial z}-K_{a}\;{\mathrm{SI}}_{N}(z\text{,}t) & \text{Otherwise} \end{array}\right)$$

(4) $${\mathrm{SI}}_{N}(z\text{,}0)=0$$

(5) $${\left(\frac{\partial {\mathrm{SI}}_{N}}{\partial z}\right|}_{z=0}={\left(\frac{\partial {\mathrm{SI}}_{N}}{\partial z}\right|}_{z=L}=0$$

The rate of absorption will be the sum of the absorption rates along the length of the intestine model:

(6) $$A(t)=\int_{z=0}^{L}K_{a}\;{\mathrm{SI}}_{N}(z\text{,}t)\mathrm{dz}$$

## Appendix B Numerical solution

The models equations were solved in MATLAB using a finite difference method. The spatial domain of the PDE was approximated using a backwards finite difference method, and the temporal domain of all the equations was solved using forward Euler method. The temporal and spatial step will be defined as Δt and Δz, respectively.

Eq. (1) thus becomes:

(1a) $${\mathrm{Stom}}_{N}(t+\Delta t)={\mathrm{Stom}}_{N}(t)-\Delta t(-\gamma {\mathrm{Stom}}_{N}(t))$$

and Eq. (3) becomes:

(3a) $${\mathrm{SI}}_{N}(z\text{,}t+\Delta t)={\mathrm{SI}}_{N}(z\text{,}t)+\Delta t\left(-\frac{\overset{¯}{u}}{\Delta z}({\mathrm{SI}}_{N}(z\text{,}t)-{\mathrm{SI}}_{N}(z-\Delta z\text{,}t))-K_{a}{\mathrm{SI}}_{N}(z\text{,}t)\right)$$

From the Von Neumann Analysis we can then define the stability of the PDE for the finite difference method used as:

(24) $$\frac{\Delta t\overset{¯}{u}}{\Delta z}⩽1$$
